# The use of a formative OSCE to prepare emergency medicine residents for summative OSCEs: a mixed-methods cohort study

**DOI:** 10.1186/s12245-021-00383-4

**Published:** 2021-10-01

**Authors:** Magdalene Hui Min Lee, Dong Haur Phua, Kenneth Wei Jian Heng

**Affiliations:** Department of Emergency Medicine, Tan Tock Seng Hospital, 11 Jalan Tan Tock Seng, Singapore, 308433 Singapore

**Keywords:** Formative, Summative, OSCE, Examination, Education, Residency

## Abstract

**Background:**

The objective structured clinical examination (OSCE) is a part of emergency medicine (EM) examinations such as the Masters of Medicine in Emergency Medicine (MMed) examination and the equivalent Member of the Royal College of Emergency Medicine (MRCEM) examination. The use of formative OSCEs to prepare EM residents for summative OSCEs has not been investigated. This study aimed to evaluate the role of formative OSCEs in preparing EM residents for the MMed and MRCEM OSCE.

**Methods:**

This was an observational, retrospective, mixed-methods cohort study. We analysed data from formative OSCEs conducted by the National Healthcare Group EM residency programme from 2013 to 2019, and from a questionnaire distributed to all residents during the study period. Residents’ formative OSCE participation and scores were compared with first-attempt summative OSCE success. Qualitative analysis of residents’ opinions on the usefulness of the formative OSCE was performed.

**Results:**

Forty-three of the 50 (86.0%) residents attended at least one formative OSCE. Of the 46 who responded to the questionnaire, 40 (87.0%) had attempted and succeeded in the MMed or MRCEM OSCE, of whom 35 (87.5%) had succeeded on the first attempt. Residents who succeeded in the summative OSCE on the first attempt tended to have higher proximate (mean = 70.6, SD = 8.9 vs mean = 64.3, SD = 10.8) and mean (mean = 67.4, SD = 7.1 vs mean = 62.8, SD = 7.3) formative OSCE scores. All 8/40 (20.0%) residents who attended more than three formative OSCEs succeeded in the summative OSCE on their first attempt. Residents’ formative OSCE scores tended to improve with successive formative OSCEs, demonstrating a positive training effect. All residents felt that the formative OSCE was useful in preparing them for the summative OSCE.

**Conclusions:**

Participation in multiple formative OSCEs was beneficial in preparing residents for the summative OSCE. The formative OSCE was useful in familiarising residents with the examination, giving them an opportunity to perform in near-examination conditions, and providing feedback to residents and faculty about their progress. Our findings may support the implementation of formative OSCEs in other training programmes to prepare learners for high-stake summative OSCEs.

## Introduction

Since the objective structured clinical examination (OSCE) was first described in 1975 [1], its use in medical education has increased. There is evidence that an appropriately designed OSCE is effective in assessing clinical competence [2–4], and OSCEs are a part of high-stakes medical examinations in many countries [5, 6].

OSCEs may be summative or formative. Summative OSCEs formally assess the outcome of learning, and are typically used in examinations to make a judgement about a candidate’s fitness to advance to the next stage of education or career. In contrast, formative OSCEs are primarily learning tools that use the format of an assessment to provide feedback to learners and guide future learning.

In emergency medicine (EM) training programmes, formative OSCEs have been used as an educational tool to train residents and assess their clinical skills [7, 8], and there are reports that OSCEs are able to predict future performance, both overall and in specific core competencies [9]. Summative OSCEs are also a component of many licencing examinations, including the Masters of Medicine in Emergency Medicine (MMed) examination, conducted by the National University of Singapore, and the equivalent Member of the Royal College of Emergency Medicine (MRCEM) examination, conducted by the Royal College of Emergency Medicine (RCEM) in the UK.

Formative OSCEs can be costly, time-consuming and labour-intensive to organise [10, 11]. It is therefore important for educators who are considering implementing formative OSCEs to understand their role in preparing learners for summative OSCEs. Previous studies done amongst internal medicine residents [12, 13], paediatrics residents [14] and medical students [15] have shown correlations between formative OSCE scores and national examination or in-training examination scores. However, studies on the effectiveness of formative OSCEs in improving medical students’ summative OSCE results have had mixed results. Due to the heterogeneity of the studies, the reasons for this are unclear. A study involving multiple formative OSCEs appeared to be more effective in improving summative OSCE pass rates [16] compared to studies involving a single formative OSCE [17, 18]. Formative OSCEs may also be more effective in improving summative OSCE scores for stations testing procedural skills rather than theoretical knowledge [19]. There have been no previous studies on the effectiveness of formative OSCEs in improving or predicting summative OSCE scores in EM, in which OSCEs typically place greater emphasis on resuscitation and procedural skills compared to OSCEs in other specialties.

We analysed 7 years of data from formative OSCEs conducted by an EM residency programme, comparing residents’ participation and performance in formative OSCEs with first-attempt success in the MMed or MRCEM OSCE, both of which will henceforth be referred to as the summative OSCE. We aimed to evaluate the role of formative OSCEs in preparing EM residents for the summative OSCE and to analyse if formative OSCE participation and performance was associated with first-attempt summative OSCE success.

## Methods

### Study design

This was an observational, retrospective, mixed-methods cohort study. Formative OSCEs have been conducted by the National Healthcare Group (NHG) EM residency programme in Singapore once or twice per year since 2013. Data were obtained from the results of formative OSCEs conducted from 2013 to 2019, and from a questionnaire distributed to NHG EM residents who had been in the programme during the study period.

### Summative OSCE

Residents are required to sit for either the MMed or the MRCEM examination at any point during junior residency, which comprises the first 3 years of a 5-year residency programme, in order to progress to senior residency. Both the MMed and MRCEM examinations contain three parts. The first is a multiple-choice test, the second comprises short answer questions and the third is an OSCE. Applicants are required to pass the first two parts before sitting for the OSCE.

The MMed OSCE consists of 15 stations, with 12 min each for three resuscitation stations and 10 min each for the other stations. Each station is preceded by 2 min of reading time. The MRCEM OSCE consists of 18 stations (16 patient encounters and two rest stations), each 7 min long and preceded by 1 min of reading time. The MMed OSCE is mapped to the competencies of years 1-3 of the emergency medicine core curriculum, Singapore, whilst the MRCEM OSCE is mapped to the competencies of years 1-3 of the RCEM emergency medicine curriculum. Both curricula are similar, and expect competence in evaluating and managing adult and paediatric emergencies seen at a general emergency department. The content of both examinations is similar, and includes the following stations: history-taking (e.g. undifferentiated headache), physical examination (involving real patients with stable pathologies, e.g. valvular heart disease), communication (e.g. breaking bad news), procedures (e.g. performing toilet and suture on a manikin), resuscitation (e.g. cardiac arrest, major trauma) and others (e.g. mass disaster response and triage). The stations involve real patients, simulated patients, task trainers and manikins. Candidates are assessed by one assessor per station using station-specific checklists. Scores for various domains, as well as a global score, are given. If a simulated patient is present, scores are calibrated with input from the simulated patient.

The MMed OSCE is standard set via the modified Angoff method [20, 21]. Candidates are required to pass at least 11 out of 15 total stations, and two out of three resuscitation stations, to pass the examination. The MRCEM OSCE is standard set via the borderline regression method [22]. One standard error of measurement is added to the identified cut-off score to calculate the pass mark. Candidates are required to achieve the pass mark and pass at least one out of two resuscitation stations to pass the examination.

### Formative OSCE

The formative OSCE is targeted at NHG EM junior residents who have yet to pass the summative OSCE. It is designed and blueprinted to be similar to the summative OSCE in format and core competencies tested. Each formative OSCE consists of 10-15 stations. The time allotted is the same as for the MMed OSCE, that is, 12 min for resuscitation stations and 10 min for the other stations, with 2 min of reading time prior to each station. The content of the stations, competencies expected and patient characteristics are designed to be similar to those of the summative OSCE. However, the formative OSCEs generally contain fewer stations than the summative OSCE, owing to logistical and manpower constraints.

The content of the stations changes in each OSCE. All cases are designed by the institution’s core faculty. The cases undergo two to three rounds of review and quality control by faculty, and standards are set via the modified Angoff method, with marking checklists and passing scores determined. Similar to the summative OSCE, residents are directly observed and scored by one assessor per station, using checklists that are designed to be similar to those of the summative OSCE, with input from the simulated patient if present. The assessors, who are members of the faculty, are trained on the assessment standards prior to the OSCE.

After the formative OSCE, a group feedback session for all participants is conducted, during which the assessors provide verbal feedback on each station. Residents are also shown their mark sheets, which contain their scores and written feedback from assessors for every station.

The number of participants in each formative OSCE varies depending on the number of residents who have yet to pass the summative OSCE. The number of formative OSCEs that a resident participates in prior to attempting the summative OSCE also varies as residents are given the freedom to decide at which stage of junior residency they attempt the summative OSCE. Residents can choose not to attend a formative OSCE if they are at work or on leave.

### Selection of participants

All NHG EM residents during the study period were included. Residents who left the programme midway were excluded.

### Data collection and processing

Data from scores for all formative OSCEs conducted during the study period were used. An online questionnaire using Google Forms was distributed via email to all current and past residents who were in the residency programme during the study period, and responses were collected over 1 month in November 2019. The data obtained from the questionnaire included whether and when residents had sat for and/or passed the summative OSCE. Residents’ perceptions of the usefulness of the formative OSCE were obtained on a 4-point Likert scale (1 = not useful, 4 = very useful), and through a mandatory free-text question on why they felt the formative OSCE was or was not useful.

An EM residency programme coordinator, who was not in the investigating team, distributed the questionnaires, collated the data, removed all identifiers and assigned identifying numbers to each participant before passing the data to the investigators. The programme coordinator checked the validity and accuracy of the data, and the investigators verified the internal consistency of the data.

### Data analysis

We compared residents’ formative OSCE participation and scores with first-attempt summative OSCE success. We also compared other factors with first-attempt summative OSCE success, namely residency year, postgraduate year and residents’ perceptions of the usefulness of the formative OSCE. Categorical variables were analysed with Pearson’s chi-square and continuous and ordinal variables were analysed with Mann-Whitney *U* tests. Qualitative analysis of residents’ opinions on the usefulness of the formative OSCE was performed to triangulate the findings. All quantitative data were analysed using IBM SPSS Statistics version 23 (IBM Corp., Armonk, NY, USA).

## Results

There were 57 EM residents from 2013 to 2019, of whom seven were excluded as they left the programme midway. Of the remaining 50, data on formative OSCE attendance and scores were available for all. Forty-six (92.0%) responded to the questionnaire.

**Fig. 1:**
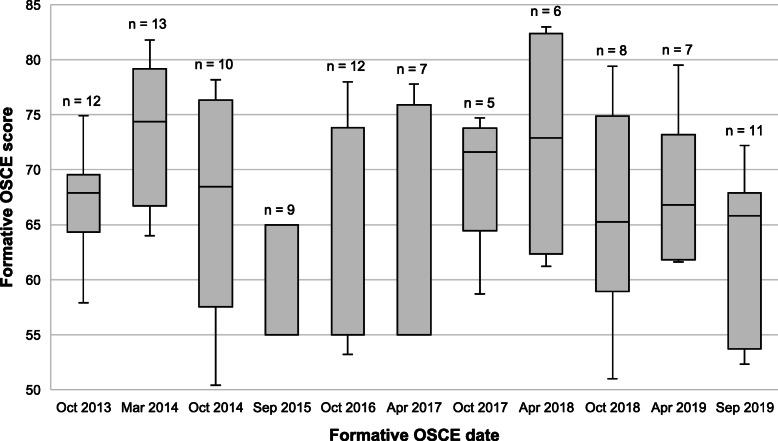
Formative OSCE scores

Forty-three of the 50 (86.0%) residents had attended at least one, and up to five, formative OSCEs (Table 1). Of the 46 who responded to the questionnaire, 40 (87.0%) had attempted and succeeded in the summative OSCE, of whom 35/40 (87.5%) had succeeded on the first attempt. The number of participants and scores for each formative OSCE are shown in Fig. 1.

**Table 1:** Formative and summative OSCE performance

Characteristic	Residents
**Attended at least one formative OSCE**	43/50 (86.0%)
**Attempted summative OSCE**	40/46 (87.0%)
**Passed summative OSCE**	40/40 (100.0%)
**Passed summative OSCE on first attempt**	35/40 (87.5%)
**Perception of usefulness of formative OSCE on a scale of 1-4**	
1-2	0/46 (0.0%)
3	10/46 (21.7%)
4	36/46 (78.3%)

**Fig. 2:**
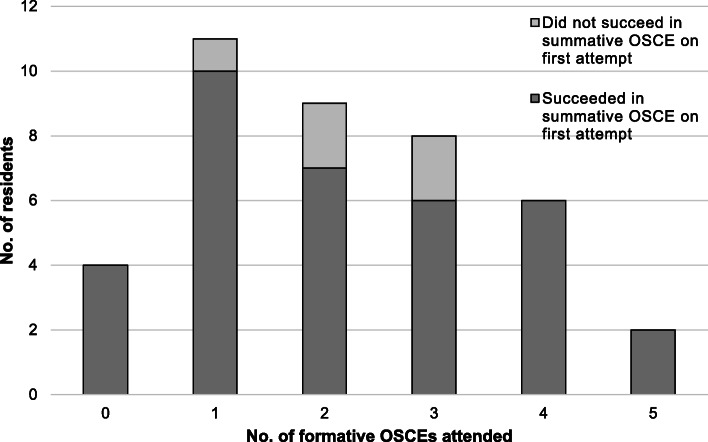
Comparison of number of formative OSCEs attended with first-attempt summative OSCE success

**Fig. 3:**
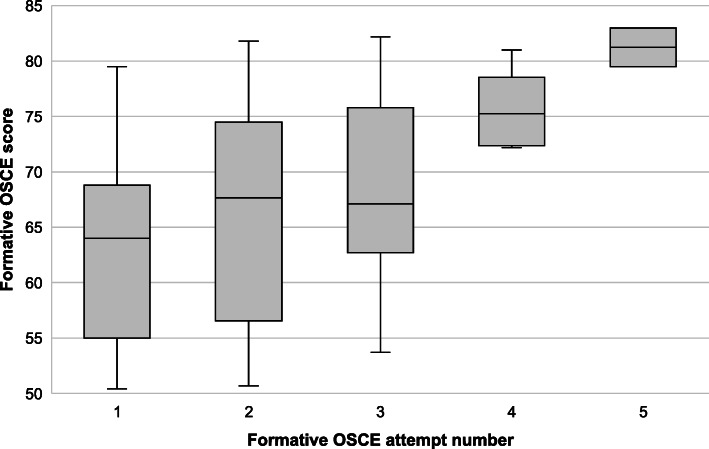
Trend of scores in successive attempts at the formative OSCE

The comparison of variables with first-attempt summative OSCE success is shown in Table 2. Residents who succeeded in the summative OSCE on the first attempt tended to have higher proximate (mean = 70.6, SD = 8.9 vs mean = 64.3, SD = 10.8) and mean (mean = 67.4, SD = 7.1 vs mean = 62.8, SD = 7.3) formative OSCE scores. Attending at least one formative OSCE was not associated with first-attempt summative OSCE success (*p* = 0.426). All 8/40 (20%) residents who attended more than three formative OSCEs succeeded in the summative OSCE on their first attempt (Fig. 2). With successive formative OSCEs, residents’ formative OSCE scores tended to improve (Fig. 3).

**Table 2:** Comparison of variables with first-attempt summative OSCE success

Variable	Total (***n*** = 40)	Succeeded in summative OSCE on first attempt (***n*** = 35)	Did not succeed in summative OSCE on first attempt (***n*** = 5)	***p***
**Attended at least 1 formative OSCE (%)**	36 (90.0)	31 (88.6)	5 (100.0)	0.426
**Proximate formative OSCE score (mean ± SD)**	66.7 ± 7.2	70.6 ± 8.9	64.3 ± 10.8	0.192
**Mean formative OSCE score (mean ± SD)**	69.7 ± 9.2	67.4 ± 7.1	62.8 ± 7.3	0.262
**Residency year when first attempted summative OSCE (mean ± SD)**	2.53 ± 1.52	2.54 ± 1.62	2.40 ± 0.55	0.833
**Postgraduate year when first attempted summative OSCE (mean ± SD)**	5.38 ± 1.46	5.29 ± 1.45	6.00 ± 1.58	0.255
**Perception of usefulness of formative OSCE on a scale of 1-4 (mean ± SD)**	3.75 ± 0.44	3.80 ± 0.41	3.40 ± 0.55	0.056

All residents felt that the formative OSCE was useful in aiding their preparations for the summative OSCE, with 36 (78.3%) rating the usefulness of formative OSCEs as 4 on a 4-point Likert scale, and the rest rating 3 (Table 1). Table 3 presents the main themes in their free-text qualitative responses on the usefulness of the formative OSCE. The most common response (24 residents, 52.1%) was that the formative OSCE helped residents to familiarise themselves with the examination format, station scenarios, timing and expectations. The next most common response (18 residents, 39.1%) was that the formative OSCE provided a good opportunity for residents to practise their skills. Examples of such skills included clinical reasoning, time management, effective verbalisation of thought processes and coping with the high pressure of an examination. The only negative response as stated by 3 residents (6.5%) was that the formative OSCE was not useful for residents who had not prepared for the examination.

**Table 3:** Most common themes in residents’ opinions about the usefulness of formative OSCEs

Response	Residents (***n*** = 46)
**Positive responses**	
Helps familiarise residents with the examination	24 (52.1%)
Good opportunity to practise examination skills	18 (39.1%)
Helps residents identify their strengths and weaknesses	9 (19.6%)
Feedback given by faculty was helpful	8 (17.4%)
Enables residents to assess their readiness to take the summative OSCE	7 (15.2%)
Aids faculty in monitoring residents’ progress	2 (4.3%)
**Negative response**	
Not useful for residents who have not prepared for the examination	3 (6.5%)

## Discussion

This is the first study evaluating the impact of formative OSCEs on summative OSCE success in an EM training programme. All residents who participated in more than three formative OSCE succeeded in the summative OSCE on their first attempt, suggesting a positive training effect where the cumulative experience gained from participating in multiple formative OSCEs was beneficial to residents taking the summative OSCE. This is supported by the upward trend in residents’ scores over successive formative OSCEs.

The qualitative responses provide an explanation for this finding, with over half of residents surveyed stating that the formative OSCE was useful in helping them to familiarise themselves with the examination format. This corroborates with previous studies [19, 23]. It has been found that unfamiliarity with the format or tested clinical competencies of an OSCE is a major factor that limits performance in the OSCE, and that learners show more consistent performance levels after taking multiple formative OSCEs [16]. In our study, repeated practice and evaluation in multiple formative OSCEs reduced the surprise element of the examination and helped to improve residents’ confidence and performance. However, attending one formative OSCE was not associated with first-attempt OSCE success, suggesting that multiple formative OSCEs were required to provide adequate exposure.

A second benefit of taking multiple formative OSCEs was the opportunity for residents to practise their examination skills in a safe training examination, as expressed by two in five residents. Formative examinations are perhaps particularly valuable in preparing for OSCEs which, unlike written examinations, test not only learners’ factual knowledge but also their abilities to interact effectively with real and simulated patients, and to synthesise and communicate information succinctly to examiners [4]. Moreover, EM OSCEs tend to place a high importance on resuscitation and procedural skills, and there is evidence that these skills are more effectively learnt through simulation than traditional forms of education [24]. This may be because these stations assess leadership, prioritisation and time-sensitive skills, and require specialised resources. They are therefore more difficult for residents to practise on their own.

In the programme, junior residents are allowed to choose when to take the summative OSCE. It is therefore useful to have an indicator to guide residents on whether they are ready to attempt the summative OSCE. Several residents felt that the formative OSCE was useful for assessing their progress and gauging whether they were ready to take the summative OSCE. This was supported by the tendency of residents who succeeded in the summative OSCE to have higher mean and proximate formative OSCE scores. Although these trends were not statistically significant, this was likely due to the small proportion of residents (5/40 residents, 12.5%) who failed the summative OSCE. This low failure rate may be partially due to residents only attempting the summative OSCE when they felt adequately prepared for it, with one of the contributing factors being their formative OSCE performance. Hence, it is of practical significance, including the cost savings of the summative examination fees in the event of failure, that the formative OSCE could provide feedback to residents about whether they were ready to take the summative OSCE, and help faculty identify outstanding residents and weaker-performing residents who required more educational intervention.

Residents commented that the formative OSCE enabled them to identify their strengths and weaknesses. Though primarily a practice exam that acts as an ‘assessment of learning’, the formative OSCE was also an ‘assessment for learning’ [25]. Previous investigations have found that testing enhances long-term retention of knowledge more effectively than repeated studying [26, 27]. By requiring active engagement from residents [28], the formative OSCE stimulated self-reflection and encouraged further studying. In addition, the incorporation of a feedback component allowed faculty members to highlight areas for improvement and give constructive advice, with residents commenting that the feedback was helpful in identifying deficits in their knowledge and skills and allowing them to learn from their peers’ mistakes. Previous studies have found that even short periods of immediate feedback can improve learners’ competencies [29].

Whilst the formative OSCE was universally considered useful, a few residents raised that the formative OSCE was of limited benefit to those who did not prepare beforehand for them. The OSCE tests ‘demonstration of learning’ on Miller’s pyramid. Learners therefore require an adequate level of prior theoretical knowledge to reap maximal benefits from an OSCE. Hence, the formative OSCE may be of limited benefit to residents who are not contemplating taking the summative examination yet and have not begun to prepare.

There were some limitations. The study was performed in a single residency programme focusing on the MMed and MRCEM examinations, and further research is needed to evaluate if the results are applicable in other training programmes or other examinations. Only a small number of residents did not succeed in the summative OSCE on their first attempt, and whilst this may be an encouragement for the programme, it limited our ability to assess factors that contributed to summative OSCE success. A control arm did not exist as most residents participated in the formative OSCE prior to attempting the summative OSCE, limiting our ability to compare the results of residents who did and did not participate in formative OSCEs.

## Conclusions

In an EM residency programme, participation in multiple formative OSCEs was beneficial to residents’ preparation for the MMed and MRCEM OSCE by familiarising them with the examination and enabling them to practise the skills required for the summative OSCE. The formative OSCE provided feedback to residents on their readiness to take the summative OSCE and helped faculty members identify residents who might require more educational intervention. Attempting more formative OSCEs had a positive training effect as evidenced by increasing scores. Our findings may support the implementation of formative OSCEs in other training programmes to prepare learners for high-stake summative OSCEs.

## Data Availability

The datasets used and/or analysed during the current study are available from the corresponding author on reasonable request.

## Abbreviations

EM: Emergency medicine
MMed: Masters of Medicine in Emergency Medicine
MRCEM: Member of the Royal College of Emergency Medicine
NHG: National Healthcare Group
OSCE: Objective structured clinical examination
RCEM: Royal College of Emergency Medicine
